# Genomic sentinel surveillance: *Salmonella* Paratyphi B outbreak in travellers coinciding with a mass gathering in Iraq

**DOI:** 10.1099/mgen.0.000940

**Published:** 2023-02-24

**Authors:** Marie Anne Chattaway, Natasha Shersby, Parisha Katwa, Kyle Adair, Anais Painset, Gauri Godbole

**Affiliations:** ^1^​ Gastrointestinal Bacteria Reference Unit, UK Health Security Agency, London, UK; ^2^​ Travel Health and IHR, UK Health Security Agency, London, UK; ^3^​ Gastro and Food Safety (One Health) Division, UK Health Security Agency, London, UK

**Keywords:** Paratyphi B, Iraq, outbreak, sentinel surveillance, genomics

## Abstract

*
Salmonella* Paratyphi B infections in England are the least common imported typhoidal infection but can still cause invasive disease. Sentinel surveillance at the reference laboratory detected an outbreak from Iraq due to reported travel history, enabling enhanced PCR testing for a quick diagnosis.

## Brief report

**Fig. 1.**
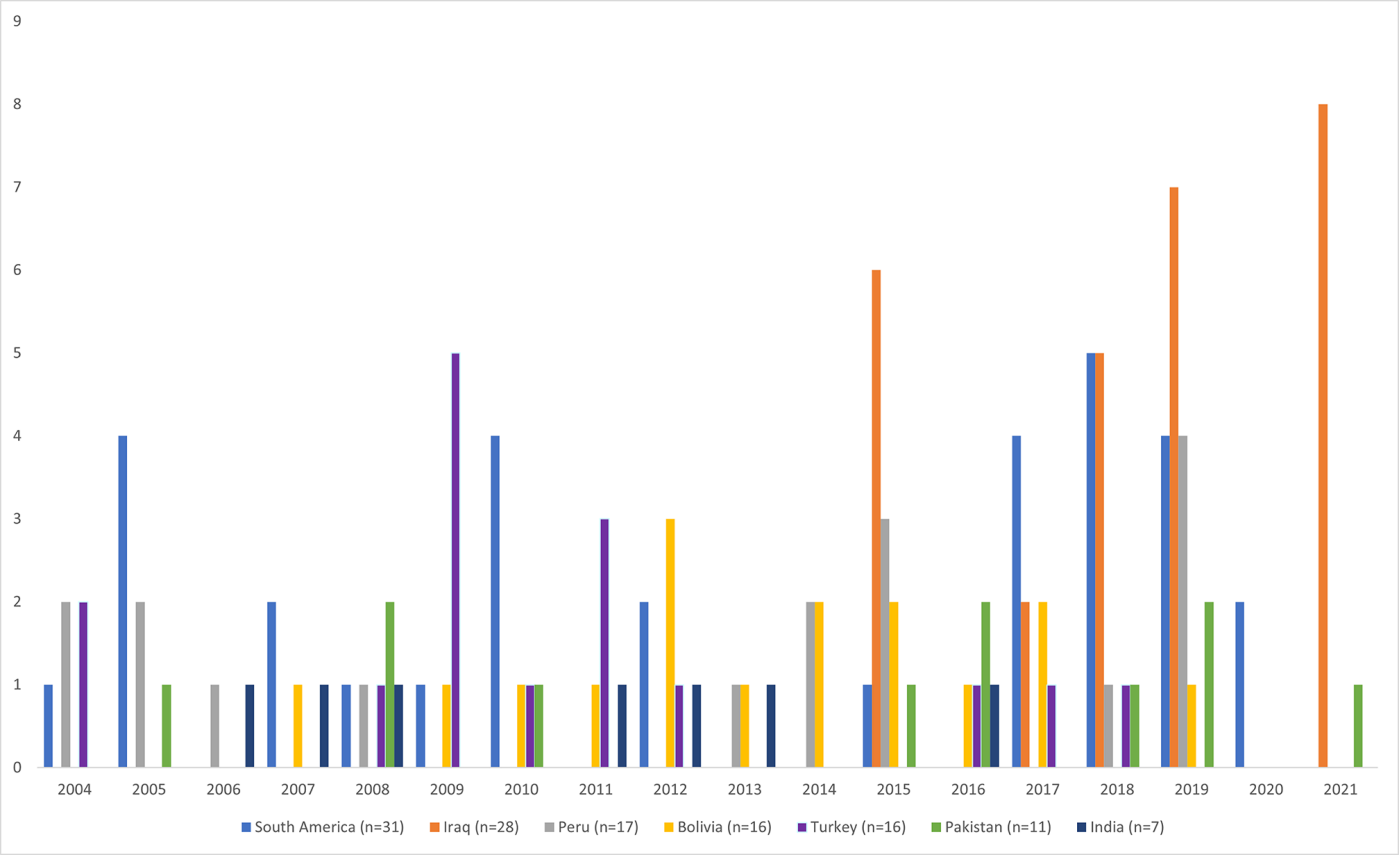
Trends of *S*. Paratyphi B cases and associated countries of travel according to sample date between January 2004 and October 2021 in England. Where a sample date was not specified the receipt date was used. The majority of travel was not stated on the request form and other countries not shown had between one and two cases over the 17 year period. South America continent travel included a variety of destinations within a case including Argentina, Bolivia, Brazil, Chile, Colombia, Ecuador and Peru.

The recent phylogenomic review of *Salmonella* Paratyphi B infections in England highlighted clades associated with travel to South America and Iraq and that routine genomic sequencing of imported cases can be used as sentinel surveillance [1]. Since developing a typhoidal PCR, the Gastrointestinal Bacteria Reference Unit (GBRU) routinely performs PCR on presumptive typhoidal *Salmonella* or strains that pose high-risk factors such as travel to endemic regions or isolate source (e.g. blood), and strains with high-risk factors are selected for PCR to provide a quick confirmatory result [2]. Isolates are then routinely sequenced for further typing characteristics such as SNP analysis [3], and publically available database platforms updated (*n*= 63 880 *Salmonella* SRA experiments as of 31 October 2021, NCBI Bioproject, PRJNA248792). Following the first COVID lockdown in March 2020, *S*. Paratyphi B cases in England dramatically dropped by 75 % from 20 cases in 2019 to five cases in 2020 (Fig. 1). In August 2021, the GBRU laboratory noticed an increase of *S*. Paratyphi B cases by molecular PCR with some request forms stating travel to Iraq. The processing criteria for typhoidal *Salmonella* (isolate from blood, travel to an endemic region or presumptive typhoidal *Salmonella *) was immediately updated (previously, the most high-risk endemic areas for typhoidal *Salmonella* included travel to India, Pakistan and Bangladesh). This ensured the PCR molecular testing of any *Salmonella* isolate referrals where request forms stated travel to Iraq, irrespective of whether the sending laboratory suspected typhoidal *Salmonella* (*n*=8), identifying a total of 11 cases to date (31 October 2021, Table 1) with 68 % being isolated from blood and likely requiring treatment. Half of the cases (6/11) were 10 years old or younger and the oldest case was 34 years old. Initial genomic data of two of these cases indicated that the strains fell into the same five-SNP single linkage cluster (2.2.30.38.135.141 .x) and that the genomic profile was fully sensitive to first-line antibiotics including ciprofloxacin. To explore the wider context, *S*. Paratyphi B (*n*=158) isolates referred to GBRU between 2004 and October 2021 were included for further genomic analysis.

**Table 1.** List of cases and reason of travel associated with *S*. Paratyphi B from England in 2020 to 2021

Reference no.	Patient	Sample date	Travel destination	Region	Date of UK departure	Date of UK arrival	Organism identified	EBG	ST	SNP address/HC5	Reason for travel	Travel summary
SRR16568878	19	28/09/2021	Iraq	Kurdistan (Sulaymaniyah)	19/07/2021	05/09/2021	*S.* Paratyphi B	5	86	2.2.30.38.135.141.819 HC5_1620	Visiting friends and relatives	Travelled abroad from UK
SRR16568937	18	01/10/2021	Iraq	Kurdistan (Sulaymaniyah)	19/07/2021	12/09/2021	*S.* Paratyphi B	5	86	2.2.30.38.135.141.817 HC5_1620	Visiting friends and relatives	Travelled abroad from UK
SRR16278007	17	20/09/2021	Iraq	Kurdistan (Sulaymaniyah, Soran)	01/06/2021	08/09/2021	*S.* Paratyphi B	5	86	2.2.30.38.135.141.818 HC5_1620	Visiting friends and relatives	Travelled abroad from UK
SRR16301621	16	16/09/2021	Iraq	Kurdistan (Sulaymaniyah, Saidsadiq)	31/07/2021	05/09/2021	*S.* Paratyphi B	5	86	2.2.30.38.135.141.823 HC5_1620	Visiting friends and relatives	Travelled abroad from UK
SRR16173692	15	12/09/2021	Iraq	Kurdistan (Sulaymaniyah)	23/07/2021	06/10/2021	*S.* Paratyphi B	5	86	2.2.30.38.135.141.823 HC5_1620	Visiting friends and relatives	Travelled abroad from UK
SRR16301627	14	17/09/2021	Iraq	Kurdistan (Sulaymaniyah)	15/08/2021	12/09/2021	*S.* Paratyphi B	5	86	2.2.30.38.135.141.826 HC5_1620	Visiting friends and relatives	Travelled abroad from UK
SRR16199731	13	12/09/2021	Iraq	Kurdistan (Sulaymaniyah)	19/07/2021	05/09/2021	*S.* Paratyphi B	5	86	2.2.30.38.135.141.819 HC5_1620	Visiting friends and relatives	Travelled abroad from UK
SRR16077458	12	09/09/2021	Iraq	Kurdistan (Sulaymaniyah)	15/08/2021	12/09/2021	*S.* Paratyphi B	5	86	2.2.30.38.135.141.819 HC5_1620	Visiting friends and relatives	Travelled abroad from UK
SRR16134638	11	05/09/2021	Iraq	Kurdistan (Sulaymaniyah)	20/07/2021	18/08/2021	*S.* Paratyphi B	5	86	2.2.30.38.135.141.820 HC5_1620	Visiting friends and relatives	Travelled abroad from UK
SRR16012276	10	26/08/2021	Iraq	Kurdistan (Sulaymaniyah)	19/08/2021	21/08/2021	*S.* Paratyphi B	5	86	2.2.30.38.135.141.818 HC5_1620	Visiting friends and relatives	Travelled abroad from UK
SRR15924201	9	18/08/2021	Iraq	Kurdistan (Sulaymaniyah)	15/07/2021	12/08/2021	*S.* Paratyphi B	5	86	2.2.30.38.135.141.817 HC5_1620	Visiting friends and relatives	Travelled abroad from UK
SRR13871367	8	11/02/2021	Non-travel	n/a	n/a	n/a	*S.* Paratyphi B	5	86	50.153.304.436.549.619.784 HC5_262694	n/a	n/a
SRR13755486	7	03/02/2021	Pakistan	Karachi	12/12/2021	23/01/2021	*S.* Paratyphi B	5	86	2.2.140.182.547.617.781 HC5_260737	Visiting friends and relatives	Travelled abroad from UK
SRR13153678	6	13/11/2020	South American continent	Bolivia, Mexico	01/04/2019	01/02/2020	* Salmonella* Paratyphi B	5	86	2.2.62.76.79.82.774 HC5_26385	Holiday-independent	Travelled abroad from the UK
SRR12887424	5	06/10/2020	Turkey	Bodrum	16/09/2020	23/09/2020	*S.* Paratyphi B	5	86	2.2.301.429.540.610.772 HC5_246822	Holiday-independent	Travelled abroad from the UK
SRR12676775	4	06/09/2020	Non-travel	n/a	n/a	n/a	*S.* Paratyphi B	5	86	2.2.62.76.79.82.769 HC5_26385	n/a	n/a
SRR11479057	3	17/03/2020	South American continent	Colombia, Peru	01/02/2021	02/03/2020	*S.* Paratyphi B	5	86	2.2.65.79.352.385.741 HC5_135240	Holiday-independent	Travelled abroad from the UK
SRR11096910	2	27/01/2020	South American continent	Bolivia (Santiago), Chile (Natale, Torres National Park), Argentina (Buenos Aires, Patagonia, Salta), Brazil (Iguazu Falls)	10/12/2019	10/01/2020	*S. Paratyphi* B	5	86	2.2.78.94.98.129.728 HC5_1857	Holiday-independent	Travelled abroad from the UK
SRR10948446	1	27/12/2019	South American continent	Chile, Argentina, Brazil	17/11/2019	22/12/2019	*S. Paratyphi* B	5	86	2.2.62.76.501.568.717 HC5_223700	Holiday-independent	Travelled abroad from the UK

Further analysis of enhanced surveillance questionnaires indicated all cases had travelled abroad from the UK to the Kurdistan region of Iraq to visit friends and relatives, with the earliest travel date 1 June to the latest return date 6 October 2021. This time period coincides with the Islamic holiday Ashura where mass gatherings of thousands of people are known to occur. None of the 11 cases who travelled to Iraq reported attending a mass gathering event. Mass gathering including religious holidays have long been a public health concern and even if travellers do not directly attend these gatherings, transmission can occur in the household or with sharing food between households. Improved public health measures, including better sanitation, are essential for preventing ongoing transmission.

**Fig. 2.**
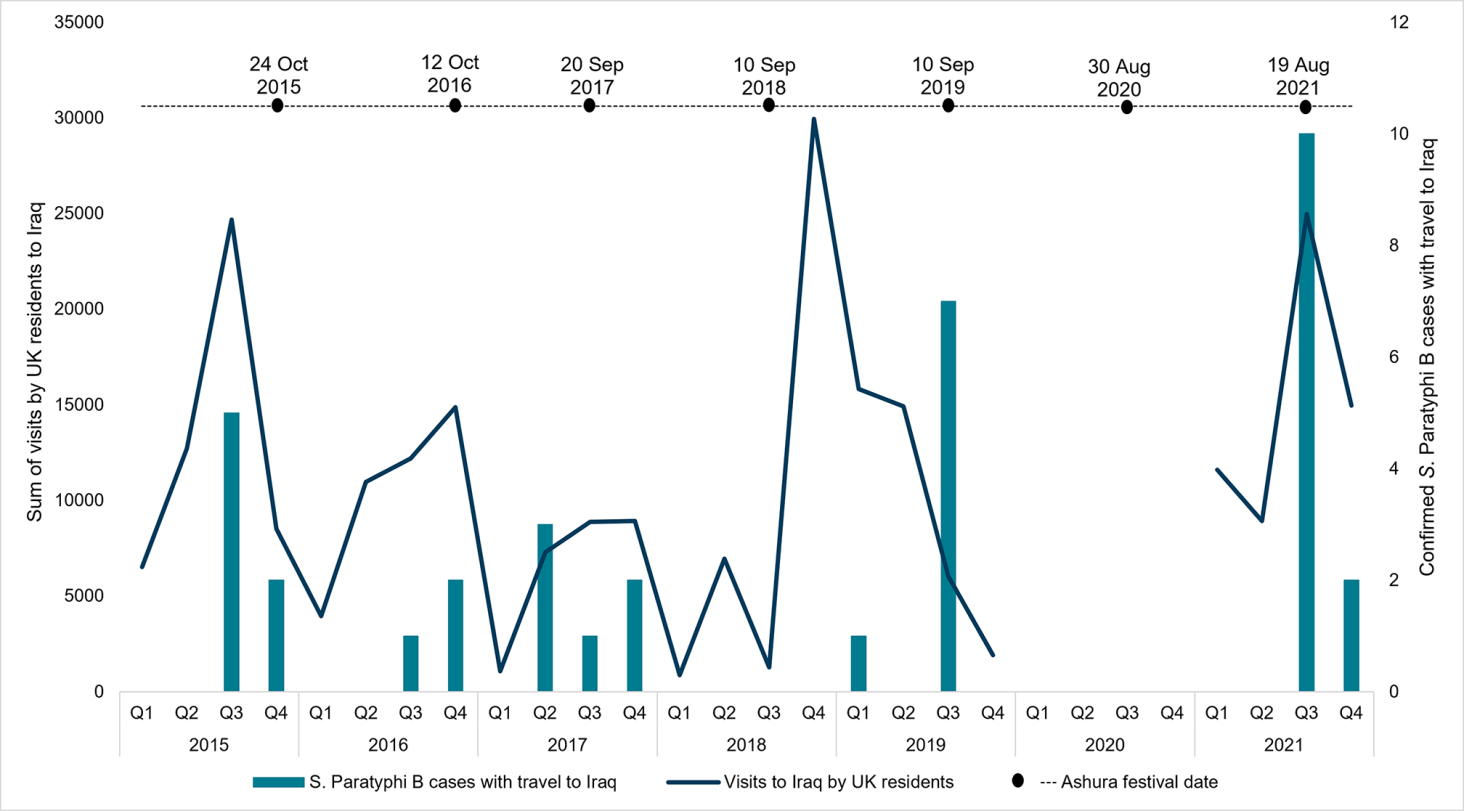
Visits by UK residents to Iraq and confirmed *S*. Paratyphi B cases with travel to Iraq against dates of the Ashura festival 2015–2021. Information on visits by UK residents is not available for 2020 as the ONS International Passenger Survey was paused in 2020 due to the COVID-19 pandemic.

Between 2015 and 2021, with the exception of 2019 and 2020, the peak of visits by UK residents to Iraq occurred either during the months of July to September (quarter 3) or October to December (quarter 4), coinciding with periods of school holidays in the UK. These dates are consistent with the increase in cases during the months of July to December (quarters 3 and 4), but in 2019 visits to Iraq declined throughout each quarter (Fig. 2). Data on visits made by UK travellers to Iraq are not available for 2020 as the ONS International Passenger Survey was paused in 2020 due to the COVID-19 pandemic. Between 2015 and 2021, excluding 2020, visits by UK residents to Iraq represented 0.7 % of travel to all countries (Office for National Statistics – International Passenger Survey). The Islamic holiday Ashura has taken place in October (2015 and 2016), September (2017 to 2019) and August (2020 and 2021) in recent years and occurs earlier each year (Fig. 2). This period is also consistent with peaks of travel to Iraq by UK residents.

**Fig. 3.**
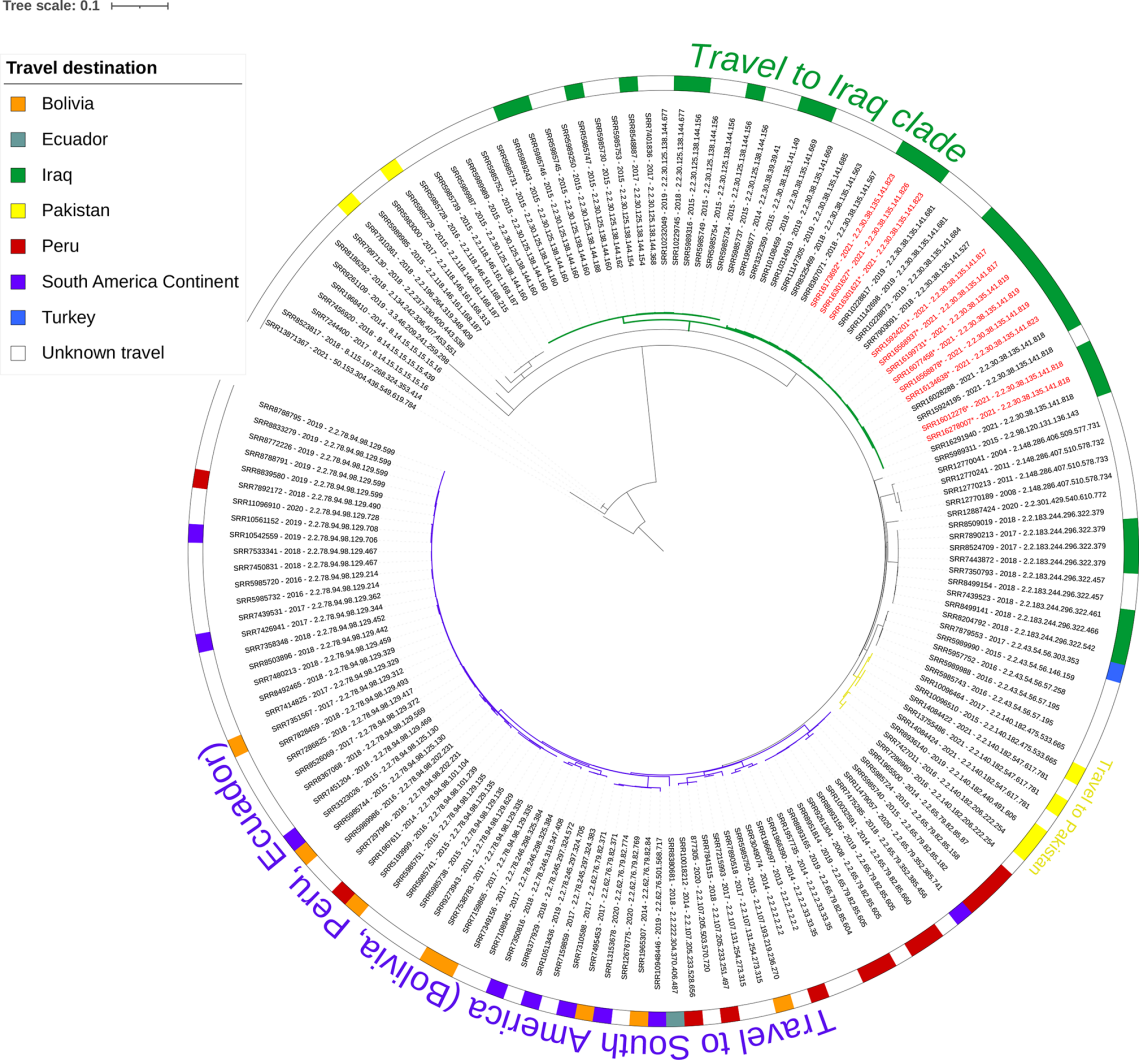
Phylogenetic tree of 153 *S*. Paratyphi B isolates referred to GBRU, 2004 to October 2021. Recent outbreak t5.141,HC5_1620 strains (red text) fall within the same cluster of endemic strains circulating in Iraq. There are distinct clades of *S.* Paratyphi B associated with South America (purple), Iraq (green) and Pakistan (yellow) since WGS began.

**Fig. 4.**
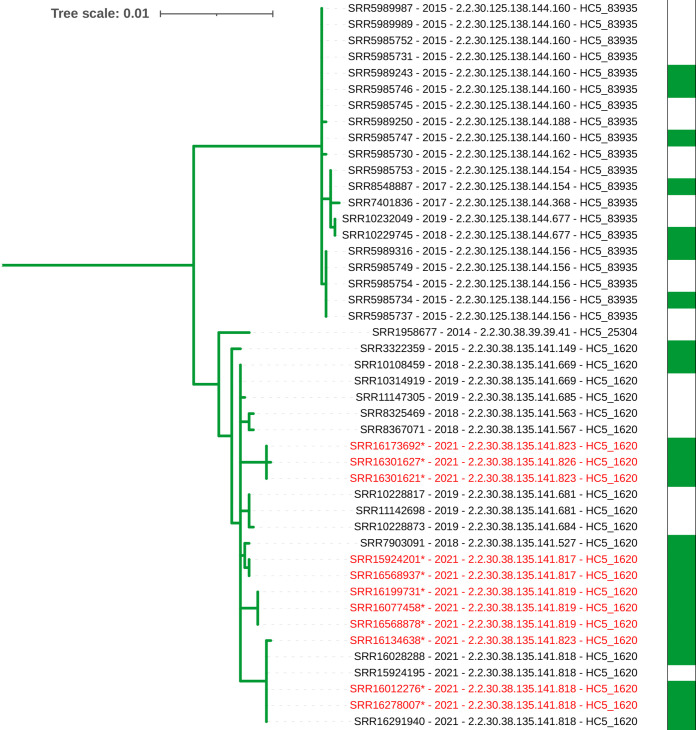
Phylogenetic clade associated with travel to Iraq. Top branch: a cluster of related cases that occurred in 2015. The recent outbreak strains from t5.141/HC5_1620 in red strains are on the bottom branch. They formed a monophyletic branch indicating sub-clusters of highly related strains (0 SNP differences). The isolates from patients with known travel to Iraq are indicated in green.

When compared within the UKHSA database of *S*. Paratyphi B genomes collected through routine surveillance of English cases, SNP typing and hierarchal clustering of core gene multilocus sequencing (cgMLST) [4] show the two initial (Table 1, patients 9 and 10) and subsequent strains (*n*=11) fall into the HC5_1620/t5.141 cluster, a clonal group circulating in Iraq [1] (Fig. 2). Outbreak strains were defined as falling within the HC5_1620 or t5.141 cluster with travel to Iraq between July and October 2021. The outbreak strains (Table 1, patients 9–19) are on average 223 SNPs from the strains that were not associated with this outbreak (Table 1, patients 1–8). The phylogeny reveals the recent Iraq isolates (*n*=11) are genetically related (0–14 SNPs, median 7 SNPs) and part of a larger clade associated with travel to Iraq (Fig. 3). A closer look at the clade associated with travel to Iraq shows two branches forming distinct clusters at different t.100 SNP level, indicating non-related strains but an endemic circulation of *S*. Paratyphi B (Fig. 4). Within the HC5_1620 /t5.141 cluster, four subclusters could be identified within the recent isolates, corresponding to strains genetically identical with the same SNP address (0 SNP differences – Table S1, available with the online version of this article). This report again highlights that although whole genome sequencing (WGS) is a highly discriminating typing method for *Salmonella* spp., where a single clone lineage is dominating in an endemic country, epidemiological data must be used to provide context in outbreak detection. In this case, the outbreak was detected by increasing numbers submitted to the reference laboratory with travel information provided on the request form. This outbreak highlighted the need to recognize emerging risks of paratyphoid fever with increased travel patterns with the relatively uncommon pathogen *S*. Paratyphi B [1]

In accordance with obligations under the International Health Regulations (2005), the UK IHR National Focal Point (NFP) informed the World Health Organization of this event, providing a summary of the situation, how the cases were detected and the measures taken in the UK. In addition, details of these cases and their travel histories in Iraq were shared by the UK IHR NFP with the Iraq IHR NFP for their awareness and to facilitate any public health actions deemed necessary.

Of the 11 *S*. Paratyphi B isolates received in this report, only 33 % indicated any travel on the request form; specifying travel on the request form is essential for assessing typhoidal risks and selecting strains for PCR to provide clinicians with a result within two working days even when typhoidal *Salmonella* is not suspected. This is the first report of the detection of an imported *S*. Paratyphi B outbreak detected in England, associated with travellers, using routine genomic sentinel surveillance.

## Supplementary Data

Supplementary material 1
